# Effective Radiosensitization of Bladder Cancer Cells by Pharmacological Inhibition of DNA-PK and ATR

**DOI:** 10.3390/biomedicines10061277

**Published:** 2022-05-30

**Authors:** Ahmed Ali Chughtai, Julia Pannhausen, Pia Dinger, Julia Wirtz, Ruth Knüchel, Nadine T. Gaisa, Michael J. Eble, Michael Rose

**Affiliations:** 1 Department of Radiation Oncology, RWTH Aachen University, 52074 Aachen, Germany; meble@ukaachen.de; 2 Institute of Pathology, RWTH Aachen University, 52074 Aachen, Germany; jpannhausen@ukaachen.de (J.P.); pia.dinger@alumni.fh-aachen.de (P.D.); jwirtz@ukaachen.de (J.W.); rknuechel-clarke@ukaachen.de (R.K.); ngaisa@ukaachen.de (N.T.G.); 3 Center for Integrated Oncology Aachen Bonn Cologne Duesseldorf (CIO ABCD), 52074 Aachen, Germany

**Keywords:** bladder cancer, DNA-PK, ATR, AZD7648, Ceralasertib, targeted therapy, ionizing radiation (IR)

## Abstract

This study aims at analyzing the impact of the pharmacological inhibition of DNA damage response (DDR) targets (DNA-PK and ATR) on radiosensitization of bladder cancer cell lines of different molecular/histological subtypes. Applying DNA-PK (AZD7648) and ATR (Ceralasertib) inhibitors on SCaBER, J82 and VMCUB-1 bladder cancer cell lines, we revealed sensitization upon ionizing radiation (IR), i.e., the IC_50_ for each drug shifted to a lower drug concentration with increased IR doses. In line with this, drug exposure retarded DNA repair after IR-induced DNA damage visualized by a neutral comet assay. Western blot analyses confirmed specific inhibition of targeted DDR pathways in the analyzed bladder cancer cell lines, i.e., drugs blocked DNA-PK phosphorylation at Ser2056 and the ATR downstream mediator CHK1 at Ser317. Interestingly, clonogenic survival assays indicated a cell-line-dependent synergism of combined DDR inhibition upon IR. Calculating combined index (CI) values, with and without IR, according to the Chou–Talalay method, confirmed drug- and IR-dose-specific synergistic CI values. Thus, we provide functional evidence that DNA-PK and ATR inhibitors specifically target corresponding DDR pathways retarding the DNA repair process at nano-molar concentrations. This, in turn, leads to a strong radiosensitizing effect and impairs the survival of bladder cancer cells.

## 1. Introduction

Bladder cancer (BC) is the ninth most common cancer worldwide, with a yearly incidence of approximately 430,000 cases, and it ranks 13th in terms of yearly mortality from cancer [1]. Histologically, bladder cancer comprises a heterogeneous group of tumors, including urothelial carcinomas (UC), and rare subtypes, such as pure squamous cell carcinomas (SCC). Nevertheless, the range of treatments available so far do not consider the broad spectrum of heterogeneity but depend rather on the stage of urothelial cancers. For localized disease, the anatomy of the bladder provides an inherent advantage, as the internal mucosal lining is accessible via a simple catheterization process. This allows for the instillation of intravesical therapies, such as chemotherapeutic agents [2] and bacillus Calmette–Guérin vaccine [3]. The current standard of care varies between the stages of disease; essentially, all >T2 or N0/N+ tumors are managed with neoadjuvant chemotherapy followed by radical cystectomy with pelvic node dissection. An organ-sparing approach for MIBC (muscle-invasive bladder cancer) is trimodal therapy (TMT), consisting of maximal transurethral resection of the bladder tumor (TUR-BT) followed by concurrent chemotherapy and radiotherapy. However, only about 30–50% of patients respond initially and most patients will progress or become refractory to treatment [4,5]. In this setting, chemotherapy is given concurrently with radiotherapy as a radiosensitizer [6]. The concept of radiosensitization is not new [7]; however, in the era of targeted therapies and immune checkpoint modulators, many clinical trials are focusing on combining radiotherapy with these new agents [8].

A special focus is on the inhibition of the DNA damage response (DDR) pathways to substantially impair the DNA repair mechanisms of malignant cells [9]. Although all the mechanisms of DNA damage repair are incredibly complex, there are, however, four main established pathways through which DNA damage is repaired: non-homologous end-joining (c-NHEJ), microhomology-mediated end-joining (MMEJ), single-strand annealing (SSA) and homologous recombination (HR). MMEJ and SSA pathways are much less studied and are often collectively known as alternative end-joining (a-EJ) pathways. HR provides the greatest fidelity because it uses sister chromatids to repair double-strand breaks (DSBs) but can only occur in the S and G2 phases of the cell cycle [10]. Both EJ pathways can occur throughout the cell cycle, although a-EJ is more active during the S phase [11]. Instead of using template strands in HR, c-NHEJ ligates two strands of DNA across a break. If HR cannot repair a DSB, the error-prone c-NHEJ becomes the dominant pathway [12]. So far, there are accumulating studies proposing radiosensitizing in various tumor entities, such as pancreatic and breast cancers, by targeting two main players in the DDR network, i.e., ataxia telangiectasia and Rad3-related kinase (ATR) and DNA-dependent protein kinase (DNA-PKcs) [13,14,15,16]:

1. ATR, along with ataxia-telangiectasia mutated kinase (ATM), plays a significant role in activating the DNA response. ATR under physiological conditions is known to protect against replication stress and to safeguard genomic stability during replication by preventing the breakage or ‘collapse’ of stalled replication forks [17]. ATR promotes the recruitment of ATRIP, the regulatory partner of ATR, thereby allowing the ATR–ATRIP complex to recognize the replication protein A bound ssDNA at DNA damage sites or stressed replication forks [18]. The ATR-dependent DNA damage repair is known to follow the HR pathway [19]. ATR initiates the S and G2 cell cycle checkpoint through CHK1 phosphorylation [15], which in turn mediates CDC25A-C phosphorylation, leading to blocking of CDK1 and CDK2 (thus preventing cell cycle progression) [20]. Inhibition of these pathways causes a dramatic increase in replication initiation, or, rather, a lack of cell cycle arrest with an eventual mitotic catastrophe due to the lack of DNA repair. Furthermore, ATR-CHK1 activation has been associated with the expression of PD-L1 expression on irradiated tumor cells in a type I IFN signaling-dependent manner [21,22].
2. DNA-PKcs, which belongs to the phosphoinositide 3-kinase (PI3K)-related protein kinase (PIKK) family, just like ATR and ATM, plays a central role in regulating c-NHEJ. c-NHEJ can repair DSBs of varying complexity, such as those with incompatible ends or damaged bases [23]. The DSB end-recognition is detected by the Ku70/80 heterodimer (Ku), which serves as a scaffold for the assembly of the components of the c-NHEJ machinery, including DNA-PKcs, the XRCC4-DNA ligase IV complex, and XRCC4-like factor (XLF) [24]. The role of DNA-PK in regulating the cell cycle is still not completely understood. However, DNA-PK demonstrates cross-talk between ATM/ATR and plays a role in checkpoint recovery, albeit in a cellular context [25,26]. DNA-PK inhibitors have also been documented to substantially enhance PD-L1 expression in irradiated cancer cells via cGAS/STING pathway activation in a p53-deficient background [27].

In the field of bladder cancer, there are several clinical trials focusing on combining chemoradiotherapy with immune checkpoint modulators [9], but there are no clinical trials focusing on DNA damage response pathway inhibitors combined with ionizing radiation to target bladder cancer. This study aims at assessing the impact of a potent and selective DNA-PK inhibitor (DNA-PKi: AZD7648) and an ATR inhibitor (ATRi: Ceralasertib) to foster radiosensitization of bladder cancer cells in vitro.

## 2. Materials and Methods

### 2.1. Cell Lines

The urothelial cell lines, UROtsa, J82 and VMCUB-1, were obtained from the American Type Culture Collection (ATCC, Manassas, VA, USA). SCaBER, a basal-type bladder cancer cell line with squamous characteristics, was kindly gifted by Prof. Wolfgang Schulz/Dr. Michèle Hoffmann (Düsseldorf University Hospital, Düsseldorf, Germany). All cell lines were cultured using DMEM (Dulbecco’s Modified Eagle’s Medium) (Sigma-Aldrich, Deisenhofen, Germany) supplemented with 10% FCS (Gibco Laboratories, Berlin, Germany), 2 mM L-glutamine, and 1% penicillin/streptomycin at 37 °C with 5% CO_2_, and successfully underwent an identity check (Multiplexion GmbH, Immenstadt, Germany) prior to the experiments. All cells and clones were regularly tested for mycoplasma infection using the PCR-based Venor^®^ GeM Mycoplasma Detection Kit (Minerva Biolabs, Berlin, Germany).

### 2.2. Small Molecule Inhibitors

The DNA-PK inhibitor AZD7648 and the ATR inhibitor Ceralasertib (AZD6738) were purchased from Selleck Chemicals (Houston, TX, USA, product codes: S8843 and S7693).

### 2.3. Ionizing Radiation (IR) Source and Setup

Irradiation was performed using a medical patient linear accelerator (LINAC). Cell culture plates containing the cells and culture media were irradiated using 6 MV X-Ray beams and placed at a maximum dose depth of around 1.5 cm. The table height was adjusted so that the distance from the source to the buildup surface was 100 cm. Another 3 cm of water equivalent material was placed on the opposite surface of the cell culture plates to allow backscatter.

### 2.4. Western Blot

Cells were cultured at a density of 3.33 × 10^4^ cells/cm^2^ and allowed to attach overnight. AZD7648 and Ceralasertib (0.1–10 µM in DMSO) were added two hours before irradiation. At a time 1 h after treatment, cells were pelleted before lysis in 50 mM Tris, 150 mM NaCl, 10 mM EDTA, 1% IGEPAL, 0.5% sodium deoxycholate, and 0.1% SDS. Protein concentrations of lysis supernatants were quantified using a Pierce^™^ BCA Protein Assay kit (ThermoFisher, Carlsbad, CA, USA). An amount of 20 µg total protein lysate was separated by SDS-PAGE with a Mini-PROTEAN Tetra vertical electrophoresis cell (Bio-Rad Laboratories, Munich, Germany), transferred to nitrocellulose membranes using a Mini Trans-Blot electrophoretic transfer cell (Bio-Rad Laboratories, Munich, Germany). The membranes were incubated in 5% *w*/*v* BSA or 5% *w*/*v* non-fat dry milk blocking buffer followed by incubation with primary antibodies: anti-ATR (Cell Signaling Technology, Danvers, MA, USA, 2790, 1:1000), anti-ATR-pTyr1989 (Abcam, ab223258, 1:1000), anti-CHK1 (Cell Signaling Technology, Danvers, MA, USA, 2360, 1:1000), anti-CHK1-pSer317 (Cell Signaling Technology, Danvers, MA, USA, 2344, 1:250), anti-DNA-PK (Cell Signaling Technology, Danvers, MA, USA, 2344, 1:1000), anti-DNA-PK-pSer2056 (BoserBio, P00645, 1:1000), anti-GAPDH (Cell Signaling Technology, Danvers, MA, USA, 8884, 1:4000), and anti-PARP (Cell Signaling Technology, Danvers, MA, USA, 9542, 1:1000).

### 2.5. Neutral Comet Assay

SCaBER cells were cultured at a density of 3.33 × 10^4^ cells/cm^2^ and allowed to attach overnight. AZD7648 and Ceralasertib (10 µM in DMSO) were added two hours prior to irradiation. Cells were pelleted and resuspended at indicated time intervals (0–6 h after irradiation) in PBS. According to the manufacturer’s instructions, a neutral comet assay was performed using the CometAssay single-cell gel electrophoresis assay kit (R&D Systems, Minneapolis, MN, USA). The cell suspension was mixed 1:10 with LMAgarose (37 °C), and 50 µL was spotted onto pre-warmed comet slides. After overnight lysis (4 °C), slides were incubated in 4 °C chilled TBE buffer (90 mM Tris-base, 90 mM boric acid, 2 mM Na_2_EDTA, pH 8.5) for 15 min and then exposed to a 21 V current for 45 min in 4 °C chilled TBE buffer. After fixation in 70% ethanol and drying, slides were stained with 1:10,000 diluted HD DNA Green Stain (Intas Science Imaging, Göttingen, Germany) for 30 min at room temperature in the dark and washed with dH_2_O afterwards. DNA damage was observed using an Axiovert 100 TV fluorescence microscope (Carl Zeiss, Oberkochen, Germany) with equipped pco.panda 26 sCMOS camera system (Excelitas PCO, Kelheim, Germany) and VisiView 4.0 software (Visitron Systems) to acquire images. For each condition, the tail moment was measured using the OpenComet, open-free ImageJ plug-in (National Institutes of Health, Bethesda, MD, USA), as a software tool providing automated analysis of comet assay images.

### 2.6. Short Term Single and Combined Drug Response Analyses

Drug response curves were performed by adding XTT (Roche Diagnostics, Penzberg, Germany), with and without IR, according to the manufacturer’s instructions. Cells were seeded in 96-well plates with cell-line-specific numbers (SCaBER: 6000 cells/0.32 cm^2^; VMCUB-1: 5000 cells/0.32 cm^2^; J82: 2000 cells/0.32 cm^2^). After 24 h, cells were treated with various doses of AZD7648 (0.01–20 µM in 0.1% DMSO) and Ceralasertib (0.01–20 µM in 0.1% DMSO) while irradiated by distinct Gy doses (2, 4, 6, 8 Gy and 0 Gy as control). After 72 h incubation, the optical density was measured using an ELISA-Reader Infinite M200 Tecan (Bio-Rad). Each assay was independently performed at least three times. The combined treatment of cells was performed analogously to the single drug assays as previously specified [28].

### 2.7. Clonogenic Survival Assay

Cells were cultured overnight at different densities to compensate for cell deaths upon different irradiation doses (0 Gy: 55 cells/cm^2^, 2 Gy: 83 cells/cm^2^, 4 Gy: 110 cells/cm^2^, 6 Gy: 138 cells/cm^2^, 8 Gy: 165 cells/cm^2^). One hour prior to irradiation, cells were treated with AZD7648 or Ceralasertib (0.25 µM) or their combination (0.125 µM). Fourteen days after treatment, cells were washed with PBS, stained with 0.25% crystal violet (Merck, Darmstadt, Germany) for 30 min, and excess staining solution was removed by washing with dH_2_O. Clonogenic survival was measured densitometrically using ImageJ. Mean surviving colonies were calculated from three technical replicates. The relative number of surviving colonies was normalized for plating efficiency for each radiation dose. After that, the surviving fraction was calculated relative to the untreated (1% DMSO) non-irradiated (0 Gy) control. Please note: Due to the spreading nature of cellular growth in the case of SCaBER and VMCUB-1, we were unable to effectively count individual colonies; this was not the case for J82 cells, which formed identifiable colonies.

### 2.8. Whole Exome Sequencing

Whole exome sequencing (WES) of wildtype cell lines (SCaBER, VMCUB-1 and J82) was performed as previously described [29] with slight modification; i.e., the Enrichment Kit to SureSelect^XT^ Human All Exon V6_r2 (Target size 60 Mb; Agilent, Santa Clara, CA, USA) was used. Raw sequencing reads were aligned against hg19 for J82 and hg38 for SCaBER, VMCUB-1 and J82, respectively. Pathogenicity was predicted by the Clinvar database.

### 2.9. Statistical Data Acquisition

Two-sided *p*-values less than 0.05 were considered significant. Logarithmic transformation, normalization (defining smallest value = 0% and largest value = 100%) and non-linear regression of raw data was performed using GraphPad prism 6 software (GraphPad Software Inc., La Jolla, CA, USA). The relative inhibition rate (100%-X^inh^) and the IC_50_ (drug concentration causing 50% inhibition) values for each cell line were determined using a “log (inhibitor) vs. normalized response—variable slope” equation (best-fit values). The results of single and combination drug assays were used to calculate the combination index (CI) with Compusyn (version 1.0) [30].

## 3. Results

### 3.1. Short-Term Application of DNA-PKi (AZD7648) and ATRi (Ceralasertib) Leads to Cell Death and Radiosensitization

Novel classes of inhibitors targeting DDR pathways, such as DN-PK and ATR, are currently being assessed in the early phases of clinical trials but have not yet been tested in bladder cancer. Thus, we sought to analyze the therapeutic impact of AZD7648 and Ceralasertib for sensitizing bladder cancer cells in relation to radiotherapy. We selected three aggressive bladder cancer cell line models which reflected different molecular subtypes, i.e., basal/squamous SCaBER, urothelial basal VMCUB-1 and non-classified J82 cells. Initially, an XTT assay was used to determine the IC50 value across the cell lines. Without ionizing radiation, the IC_50_ doses for each drug, i.e., AZD7648 and Ceralasertib, were broadly similar across *n* = 3 cell lines, ranging from 6.77 to 10.57 µM (AZD7648) and from 1.70 to 3.88 µM (Ceralasertib), respectively.

**Figure 1:**
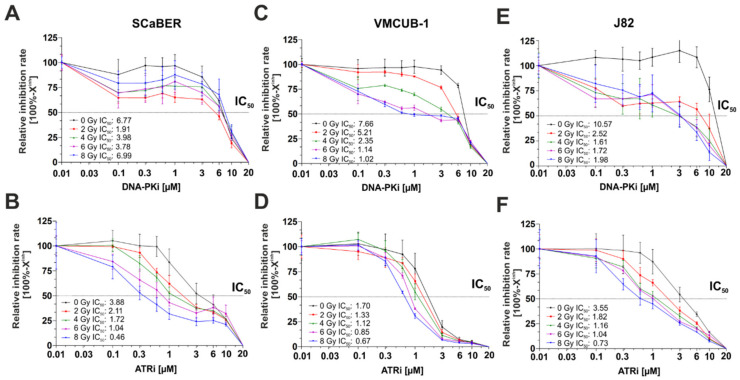
IC_50_ values of ATR and DNA-PK inhibitors in relation to radiation dose and cellular background. Short-term single drug response analyses applying DNA-PK and ATR inhibitors upon different radiation doses (0 Gy, 2 Gy, 4 Gy, 6 Gy, 8 Gy) on squamous SCaBER (**A**,**B**), urothelial VMCUB-1 (**C**,**D**) and J82 (**E**,**F**) bladder cancer cell lines. Semi-logarithmic plots show drug response curves (relative inhibition rate = 100% − X^inh^) of urothelial bladder cancer cell lines. Drug response was determined using XTT following 72 h incubation with indicated drug concentrations and radiation doses. Dotted line and stated relative IC_50_ = drug concentration causing 50% inhibition. Drug response curves represent means from at least *n* = 3 independent experiments.

We then analyzed IC_50_ values across a range of radiation doses. There was a shift in the IC_50_ towards a lower value as the radiation dose exposure increased (Figure 1A–F). Essentially, a radiosensitizing effect was verified, although, in a short-term setting (72 h), there is an overestimation of cell survival due to the presence of metabolically active cells that will eventually die [31]. Except for SCaBER and J82 cells, upon DNA-PKi treatment, the calculated IC_50_ value for DNA-PKi and ATRi decreased steadily with increasing IR dose. SCaBER was the most sensitive cell line tested upon ATR inhibition (Ceralasertib: IC_50_ = 0.46 µM at 8 Gy), while VMCUB-1 was highly sensitive upon DNA-PK inhibitor treatment (AZD7648: IC_50_ = 1.02 µM at 8 Gy). In SCaBER and J82, the lowest IC_50_ value was achieved for AZD7648 at 2 Gy (1.91 µM) and 4 Gy (1.61 µM), respectively. We finally analyzed UROtsa as a model of normal urothelial cells upon single DNA-PKi and ATRi treatment without IR and across a range of radiation doses (Supplementary Figure S1). Without drug application, UROtsa showed IC_50_ values upon IR comparable with that of the cancer cell lines. Interestingly, the normal UROtsa cells were much more resistant to DNA-PKi and ATRi when exposed to high dose IR (AZD7648: IC_50_ = 6.74 µM at 8 Gy and Ceralasertib: IC_50_ = 4.35 µM at 8 Gy).

### 3.2. Pharmacological Inhibition of DNA-PK and ATR Effectively Blocks Downstream Signaling Involved in DDR

**Figure 2:**
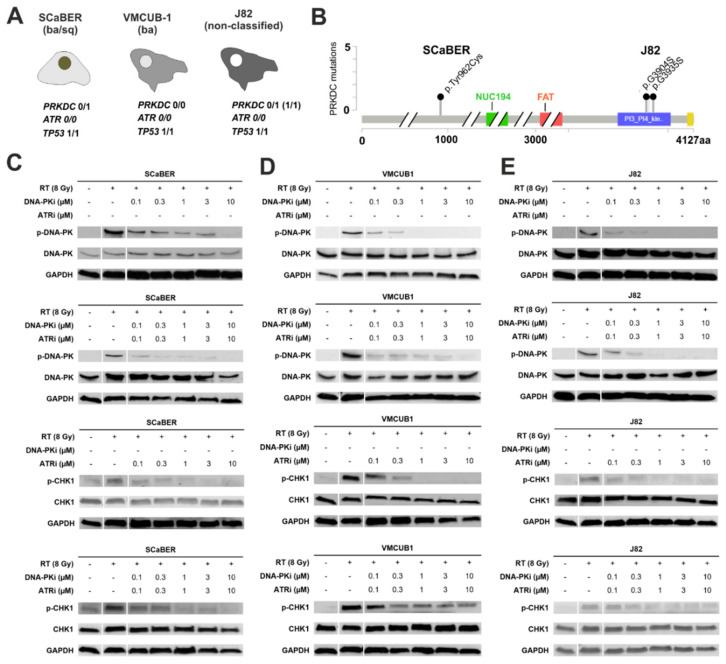
Inhibition of DNA-PK and ATR impairs downstream DDR signaling of different bladder cancer cell line models. (**A**,**B**) In vitro models used for drug response studies. NGS-based exome sequencing revealed a monoallelic PRKDC mutation of unknown functional significance in squamous-like SCaBER and two alterations in urothelial J82 cells. The urothelial basal-type VMCUB-1 cell line showed no PRKDC alteration. The ATR wild-type and biallelic TP53 mutations were present in all cell lines. (**B**) The type and position of the monoallelic *PRKDC* (DNA-PKcs) alterations of SCaBER and J82 cells are illustrated in a lollipop plot. The mutations found in SCaBER and J82 are of unknown significance. In J82 cells, the mutation of DNA-PKcs was located in the PI3_PI4 kinase domain (p.G3904S) close to the predicted binding site of AZD7648, potentially involving interaction with the amino acids at the positions Leu3751, Arg3737, Trp3805, Leu3806, Asn3926, and Ile3938 [32]. The identified mutation of DNA-PKcs in SCaBER cells was located at the N-terminal to the FAT domain, which is known to comprise a predominantly helical solenoid HEAT-repeat domain of variable length that mediates protein-protein interaction [33]. (**C**–**E**) Phospho-western blots were performed to assess activation of DNA-PK and ATR mediated pathways upon irradiation (radiation dose = 8 Gy), as well as single and dual DNA-PK/ATR inhibition, in SCaBER, VMCUB-1 and J82 bladder cancer cells. Radiation of cells triggered activation of both DDR pathways (DNA-PK and ATR). In turn, phosphorylation of DNA-PK (Ser2056) and CHK1 (Ser317) was effectively blocked upon single/dual treatment of increased concentrations of AZD7648 and Ceralasertib. Please note: For the ATR pathway, the activation of downstream target CHK1 (Ser317) was analyzed since the phosphorylation of ATR at position Thr1989 was not significantly altered upon AZD6738 (Ceralasertib) treatment (see Supplementary Figure S3). ‘+’ and ‘−’ refers to the exposure or lack thereof to IR or drug.

NGS-based exome-sequencing provided detailed information regarding mutations in DDR genes, in particular for our targets *PRKDC* and *ATR*, as well as *TP53* (Figure 2A,B). For *PRKDC*, encoding the catalytic subunit of DNA-PKcs, a monoallelic single-nucleotide variation was shown to be present in squamous-like SCaBER (p.Tyr962Cys, not reported so far) and two different monoallelic single nucleotide variations in urothelial J82 (p.Gly3935Ser, p.Gly3904Ser, uncertain significance) cancer cells. The urothelial basal-type VMCUB-1 cell line showed no *PRKDC* alterations. All three cancer cell lines were characterized by an *ATR* wildtype gene but possessed biallelic *TP53* mutations. In SCaBER and VMCUB-1 cells, *TP53* alterations were verified as pathogenic variants (SCaBER: p.Arg71Leu and p.Arg110Leu; VMCUB-1: p.Arg16His, p.Arg175His, p.Arg43His, p.Arg136His). The *TP53* mutation (p.Glu271Lys) confirmed in J82 was of uncertain significance since there are conflicting interpretations of pathogenicity (see Supplementary Table S1). The normal urothelial cell line UROtsa showed no *TP53* mutation (data not shown).

Prior to the experiments, we confirmed DNA damage triggered by various doses of IR (2, 4, 6, 8 Gy). As expected, higher radiation doses caused increased levels of DNA damage as visualized by a comet assay (see Supplementary Figure S2). Subsequently, selective inhibition of the DNA-PK and ATR pathways by AZD7648 and Ceralasertib treatment was confirmed in bladder cancer cell lines (Figure 2C–F). While the irradiation of cells (8 Gy) led to strong activation of DNA-PK and ATR (from 0% up to 100%), applied drugs (AZD7648 and Ceralasertib) caused a dose-dependent blockade of protein phosphorylation and associated DDR pathways depending on the given cell line. For instance, DNA-PKi treatment reduced DNA-PK activation at low doses (0.1 µM), i.e., remaining phosphorylation ranged between 17.8% and 46.2%, compared to the 8 Gy control (=100%). At 10 µM AZD7648, activation of DNA-PK was completely abolished, similar to CHK1 activation upon ATR inhibitor (ATRi) treatment. The ATR downstream target CHK1 (Ser317) served as a surrogate marker to assess inhibition of the ATR pathway since the phosphorylation of ATR at position Thr1989 was not helpful (see Supplementary Figure S3). For relative quantification of DNA-PK and CHK1 activation upon individual and combined AZD7648 and Ceralasertib treatment, see Supplementary Figure S4.

### 3.3. Pharmacological Inhibition of DNA-PK and ATR Impedes DNA Damage Response after Exposure to Ionizing Radiation

**Figure 3:**
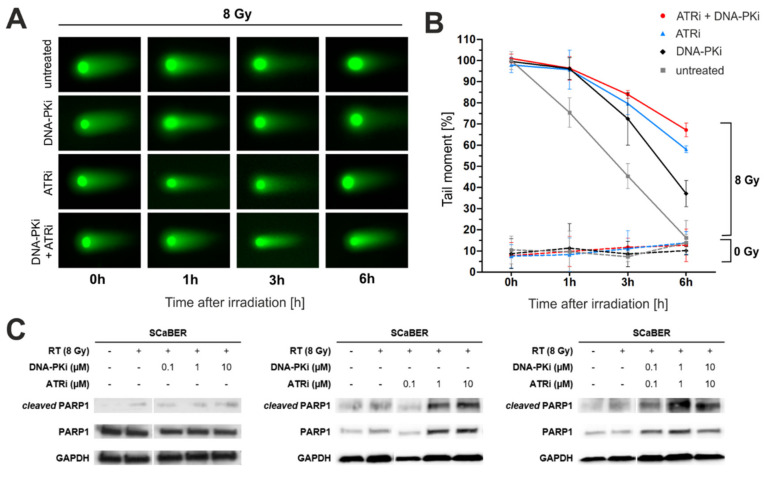
Blocking DNA-PK and ATR activity retards DNA repair of radiation-induced DNA damage in squamous bladder cancer cells. (**A**) Representative immunofluorescence images of stained nuclear tails are shown for each time point and treatment option (i.e., single DNA-PK and ATR inhibitor treatment (both: 10 µM), combined treatment application of DNA-PK and ATR inhibitors, and non-treated cells (controls)). A neutral comet assay was performed and images were obtained with 20× original magnification. (**B**) Semi-quantitative analyses of the tail moment of SCaBER cells upon combined irradiation (8 Gy) and DNA-PK/ATR DDR pathway inhibition illustrating a retarded DNA repair response. Overall *n* = 5493 individual cells (35–148 cells/sample) with corresponding tails were analyzed by ImageJ. The time point 0 h was used for normalization. The calculation of the tail moment of non-irradiated cells but upon DNA-PK/ATR treatment was considered as a control to exclude drug-induced DNA damage. Curves of tail moments represent means from at least *n* = 2 independent experiments (except for dotted control lines). (**C**) Western blots were performed to assess the cleavage of PARP1 as an apoptotic marker with and without irradiation (dose = 8 Gy) as well as upon single and dual DNA-PK/ATR inhibition in SCaBER bladder cancer cells. GAPDH was used as a loading control.

Next, we showed substantially blocked DNA repair upon DNA-PKi and ATRi treatment in squamous SCaBER cells by calculating the dimension of tail moments of comet assays 1–6 h after IR (Figure 3A). Interestingly, the dual treatment of DNA-PKi and ATRi was most efficient in retarding the DNA damage response (DNA damage remaining after 6 h: 67.02%) followed by individual ATRi (DNA damage remaining after 6 h: 58.81%) and DNA-PKi (DNA damage remaining after 6 h: 33.99%) application (Figure 3B). During this period, SCaBER cells without drug treatment repaired more than 85% of damaged DNA lesions (i.e., DNA damage remaining after 6 h: 14.52%). Effects of DNA-PKi and ATRi alone (i.e., without ionizing radiation) on DNA, potentially causing damage or interfering with DNA damage repair, could be excluded. We then analyzed the cleavage of PARP1 as an apoptotic marker [34] in SCaBER cells after single and dual DNA-PK1 and ATRi treatment with and without IR (Figure 3C). Cleaved PARP1 was predominant upon higher doses of ATRi and combined DNA-PKi-ATRi application, while single DNA-PKi treatment showed only a mild effect on PARP1 activation.

### 3.4. AZD7648 and Ceralasertib at Nano-Molar Concentrations Drastically Reduce Clonogenic Survival after Radiation Exposure

**Figure 4:**
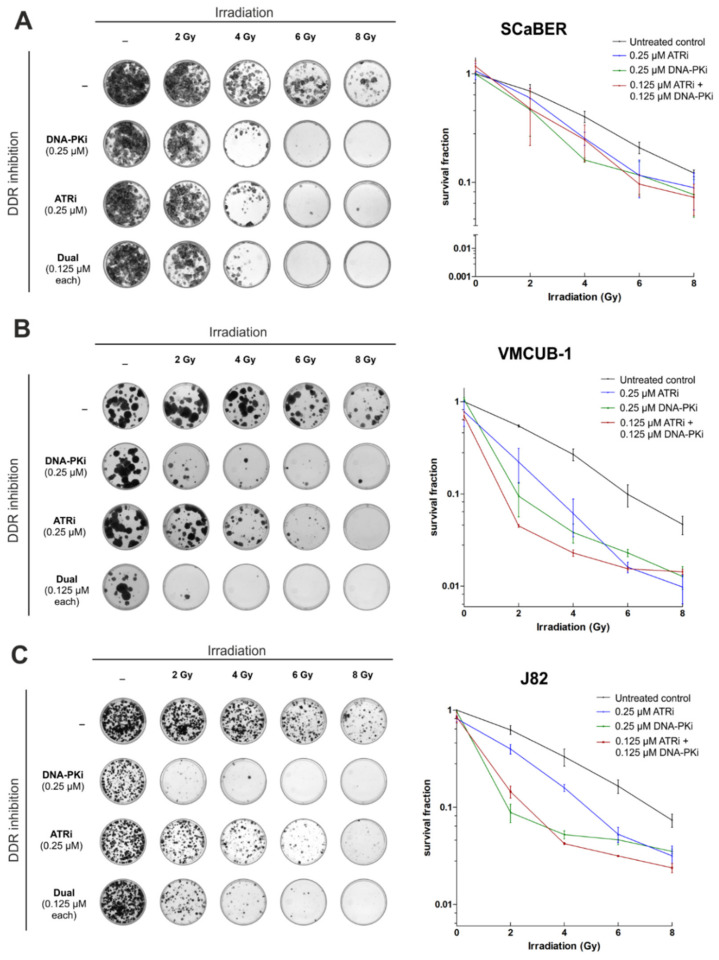
Long-term observation shows a clear inhibition of clonogenic survival of bladder cancer cells after radiation exposure. A clonogenic survival assay was performed to reveal colony formation upon pharmacological single and dual DNA-PK and ATR inhibition with irradiation (2–8 Gy) using SCaBER, VMCUB-1 and J82 bladder cancer cells. Colony growth was assessed for SCaBER (**A**), VMCUB-1 (**B**) and J82 cells (**C**) 14 days after AZD7648 and Ceralasertib treatment in combination with different radiation doses as indicated. DMSO served as the negative control. Left side: Representative images of grown colonies are shown. Right side: Survival fractions (clonogenic potential) are densitometrically illustrated for each cell line. Colony survival curves represent means from at least *n* = 2 independent experiments.

Subsequently, we aimed to show whether DNA-PK and ATR inhibition could radiosensitize bladder cancer cells. A clonogenic survival assay was performed over 14 days. SCaBER, VMCUB-1 and J82 bladder cancer cells were individually treated with either DNA-PKi (0.25 µM) or ATRi (0.25 µM), as well as in combination (0.125 µM each), upon ionizing radiation of various doses (2, 4, 6, 8 Gy). The results are illustrated in Figure 4A–C.

Overall, pharmacological inhibition of both DNA-PK and ATR caused decreased cell survival upon IR compared to controls without drug treatment. DNA-PKi, and a combination of DNA-PKi and ATRi, radiosensitized SCaBER, VMCUB-1 and J82 cancer cells most efficiently, i.e., the radiation dose was reduced by 2–4 Gy to achieve comparable results of induced cell death. In VMCUB-1 cells, a shift in sensitizing was observed up to 6 Gy. Single DNA-PKi treatment impaired cell survival (>90%) at low radiation doses (2 Gy) with strong efficacy, whereas single ATRi application was less effective at lower radiation doses in this long-term setting. At the end, single colonies still survived upon both treatment approaches with individual application of drugs, i.e., DNA-PKi or ATRi at 8 Gy IR. A complete remission of colony growth was only demonstrated in all cell lines upon a dual treatment approach (+IR) suggesting suppression of bypass mechanisms which otherwise could rescue cells.

### 3.5. Combination of AZD4678 and Ceralasertib Shows Potential Synergism Depending on Cell Line and Drug-/IR-Dose

**Figure 5:**
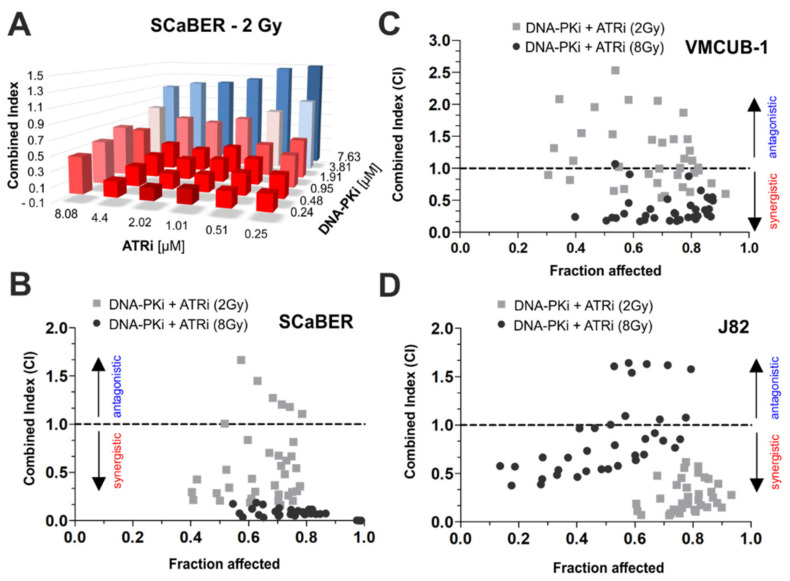
Dual ATR and DNA-PK inhibition mediates short-term synergistic effects depending on radiation dose and cellular background. The combination index (CI) was calculated in order to assess the combined effects of drugs (CompuSyn software, v.1.0). (**A**) 3D graph illustrates CI results for combined application of dual DNA-PK and ATR inhibition upon low dose irradiation of SCaBER cells. Drugs were used at concentrations of 4 × IC_50_, 2 × IC_50_, 1 × IC_50_, 0.5 × IC_50_, 0.25 × IC_50_, 0.125 × IC_50_ of each drug for SCaBER. CI = 1: additive effect, CI < 1: synergistic effect (red), CI > 1: antagonistic effect (blue). (**B**–**D**) Graphs illustrating CI results for cell fractions affected by dual application of DNA-PK and ATR inhibitors in combination with two distinct radiation doses (low 2 Gy and high 8 Gy) on SCaBER (**B**), VMCUB-1 (**C**) and J82 (**D**) cells after 72 h. CI data represents means from at least *n* = 3 independent experiments.

Based on the calculated IC_50_ values, drug-response assays with combinations of AZD4678 and Ceralasertib were performed for SCaBER, VMCUB-1 and J82 cells (Figure 5A–D). A dual drug effect was determined by calculating the combination index (CI) as a non-constant combination following the Chou–Talalay method [30]. Detailed CI results associated with applied drug concentrations upon 2 Gy IR are shown, for example, for SCaBER in Figure 5A. Strong synergism was observable at concentrations lower than the IC_50_ values of both drugs (AZD7648 and Ceralasertib) associated with CI values ranging between 0.16 and 0.68. CI values in the range of 0.67–1.67 were calculated at higher concentrations of DNA-PKi, reflecting a shift towards antagonistic effects. Figure 5B–D demonstrates the fraction affected (being equivalent to the cell death rates) upon both combined treatment and 2 Gy or 8 Gy IR. Similar to SCaBER, a dose-dependent range of CI was shown for J82 upon 8 Gy IR, i.e., dual treatment with DNA-PKi and ATRi caused either synergism at low concentrations or antagonism at high concentrations. For VMCUB-1 cells, we observed a heterogeneous pattern of synergic and antagonistic effects. In turn, combined DNA-PKi and ATRi treatment revealed an unambiguous synergism for all tested combinations in SCaBER at 8 Gy (CI range: 0.001–0.185) and in J82 at 2 Gy (CI range: 0.069–0.618).

## 4. Discussion

Pelvic radiotherapy is known to cause unavoidable exposure of the bladder, urethra, bone marrow and distal ureters to radiation which subsequently leads to many inevitable radiation-associated complications [35]. To improve the therapeutic ratio of potential bladder cancer treatment, we focused on DNA repair pathways known to affect resistance to DNA-damaging chemotherapy and radiotherapy [36]. We used novel small molecule inhibitors targeting key proteins of DNA damage response pathways [37], such as DNA-PK, as an emerging therapeutic target in cancer [38]. Similar compounds of these novel classes of drugs targeting ATR and DNA-PK have already demonstrated a radiosensitizing effect in various types of cancer cells [9,13,14,39,40,41]. Previously, Dillon et al. studied the ATR inhibitor AZD6738 (Ceralasertib) in DNA-PK deficient NOD SCID gamma mice, demonstrating an appropriate tolerance of this drug [42]. Fok and colleagues confirmed that AZD7648 is a potent and selective DNA-PK inhibitor in various cell lines [16]. To date, the impact of DNA-PK and ATR inhibition on bladder cancer cells has remained unknown. Herein, we provide functional evidence that treatment with both DNA-PKi (AZD7648) and ATRi (AZD6738; Ceralasertib) resulted in radiosensitization of bladder cancer cells of various histological/molecular subtypes, i.e., in squamous (SCaBER), in urothelial basal (VMCUB-1) and in urothelial non-classified (J82) cells.

The three cell line models used in this study are characterized by a *TP53* point mutation of known pathogenic, or at least uncertain, significance. Mutations in *TP53* are common genetic alterations in bladder cancer development, especially for muscle-invasive cancers [43]. Overall, bladder cancer is affected by a high number of genetic alterations in genes of the DDR network [44]. Thus both, the biallelic mutation in *TP53* identified in all three cell lines, as well as the mono-allelic SNVs in *PRKDC* identified in SCaBER and J82 cells, reflect typical and frequent alterations of bladder cancers.

In this molecular context, a selective blockage of the DDR pathways orchestrated by DNA-PK and ATR could be confirmed in this study on a molecular and cellular level. Overall, individual and dual treatment with ATRi and DNA-PKi radiosensitize bladder cancer cells most likely independently of their functional p53 status (at least this is the case for VMCUB-1 and SCaBER cell lines). Despite the mutational status of *TP53* in all three cell lines, it seems that all cells can undergo apoptotic cell death. Moreover, we showed that single pharmacological inhibition of ATR (by Ceralasertib) or DNA-PK (by AZD7648), as well as their combination, did not result in significant DNA damage (even at a high concentration of 1 µM) as assessed with the comet assay. However, when DNA damage was induced by ionizing radiation, repair mechanics were strongly retarded. In our experiment, the dual treatment of SCaBER cells with DNA-PKi and ATRi caused the strongest repression of DNA repair mechanisms. Consistent with these data, we confirmed increased apoptosis in SCaBER cells treated with both drugs. The lack of DNA damage by inhibitor exposure alone highlighted that these molecules would be unlikely to cause harm to cells, especially at even lower concentrations, thus potentially making them safe to use with respect to their ability to cause DNA damage, perhaps even as intravesical therapy. The ionizing radiation-induced damage and the resulting retardation of repair due to the inhibitors essentially provides a targeted approach to increase genomic instability in tumor cells. With reduced options to compensate for the missing DNA repair mechanisms, this then eventually leads to cell death. This notion was further supported by our normal UROtsa model, since these normal cells were much more resistant to ATRi and DNA-PKi at high IR doses, suggesting putative compensatory pathways allowing bypassing of ATR and DNA-PK inhibition.

However, the targeted retardation of DNA damage repair itself does not guarantee cellular death. Therefore, we used short-term and long-term assays to determine if the observed damage would eventually result in a radiosensitizing effect. The initial choice of using a metabolic assay (XTT) was used to determine the IC_50_ value of each drug and to assess a potential synergistic effect of combining each inhibitor with ionizing radiation. The general trend of lower IC_50_ values with increasing radiation doses was expected and observed with a few exceptions. An 8 Gy of IR dose shifted the IC_50_ to 6.99 µM for the SCaBER cell line and a similar event was found also for the J82 cell line where the 8 Gy dose also shifted the IC_50_ value to 1.98 µM. We could not explain this phenomenon; however, the data could point to a mechanism that results in an observation of radioresistance, which leads to prolonged cell survival in a short-term observation setting.

Using a long-term colony formation assay, we showed that nano-molar concentrations of AZD7648 and Ceralasertib resulted in a lower clonogenic survival after 14 days across all three cell lines. Low IR doses (2–4 Gy) effectively killed cells, while a complete death of colonies was achieved at higher IR doses (6–8 Gy). Across all three cell lines, AZD7648 alone was equally effective in reducing the number of surviving colonies compared to dual drug treatments. However, this data must be interpreted in the context of the concentration used for single (250 nM) and dual treatments (125 nM of each). Based on our data, we observed that DNA-PK inhibition with AZD7648 was more effective than ATR inhibition. Currently, it is not clear why AZD7648 was more effective in killing cells, even though we observed much more pronounced repair retardation kinetics in the case of Ceralasertib from the comet assay. An explanation could be that a slower repair process alone does not automatically imply insufficient repair, i.e., genetic instability. The recent report from Hafsi and colleagues demonstrated that combining Ceralasertib (AZD6738) and KU-0060638 (DNA-PKi) at 125 nM had a stronger radiosensitization effect than either drug individually at 250 nM. In contrast, our data from the clonogenic assays did not show an additive effect for all three cell lines. We observed higher killing effectiveness when both inhibitors were combined with IR in the case of VMCUB-1 but not for SCaBER and J82. However, this should also be interpreted in the context of the drug dosing.

Synergistic outcomes of dual drug treatment were further evaluated by calculating CI values in a short-term XTT assay-based setting. Combined DNA-PKi and ATRi treatment showed an unambiguous synergism for all tested combinations in SCaBER and J82 cells upon specific IR doses (SCaBER: 8Gy and J82: 2Gy). In addition, a drug concentration-dependent antagonism has been shown in VMCUB-1. Since synergisms are thought to be a physicochemical mass-action law issue of the drug-protein interaction [45], different affinities of both drugs to their targets may foster the concentration-dependent outcomes. Thus, not every combination is necessarily useful. In turn, antagonisms of combined DNA-PKi and ATRi treatment could be understood in a mechanistic context since dose-dependent interactions between ATM, ATR and DNA-PKcs have been found for two key aspects of the DNA damage response: DSB end-resection and G2-checkpoint activation [15,26]. Mladenov and colleagues recently demonstrated that ATM and ATR epistatically regulate resection at low IR doses associated with low DSB-numbers in the genome, i.e., inhibition of either ATR or ATM activity fully suppresses DNA repair. At high IR doses inducing high amounts of DSBs, the tight ATM/ATR coupling relaxes while DNA-PKcs integrates with the ATM/ATR module by regulating resection at all IR doses. Hence, the underlying mechanisms of the observed time-, drug-dose- and IR-dose-dependent synergistic and antagonistic effects of single and dual treatment seem complex and require in-depth study in the future. Another recent publication from the group discusses the interaction of DNA-PK, ATM and ATR with respect to the IR dose and cell cycle phases [25]. They state that all three integrate together in response to the amount of DNA damage caused by IR depending on the state of the cell cycle. Another aspect to consider is the intrinsic dysregulation of DDR pathways due to mutated DDR genes. We could not exclude the possibility that the presence of a single mutated *PRKDC* allele in SCaBER or J82 cells may affect the pharmacological inhibition (also the pharmacodynamics) of the c-NHEJ main mediator DNA-PK. Although we do not know the functional significance of the identified *PRKDC* mutations, an amino acid shift in J82 cells at the PI3_PI4 kinase domain (p.G3904S) close to the predicted binding site of AZD7648 (Leu3751, Arg3737, Trp3805, Leu3806, Asn3926, and Ile3938) [32] might diminish the affinity of drug-target interactions.

## 5. Conclusions

Our data revealed an effective radiosensitization of invasive bladder cancer cells of various subtypes, including SCC-like, by individual and dual DNA-PK and ATR inhibition. Since squamous bladder cancers have been associated with poor response to chemotherapy [46], radiation-based therapies combined with DDR inhibition might be a promising approach for future strategies to reduce applied drug and IR doses, thus helping to prevent cytotoxic and radiotherapeutic adverse effects for BC patients. Bearing in mind that bladder cancers are characterized by frequent genetic [44] and epigenetic alterations [47] in genes of DDR pathways potentially making cancer cells vulnerable to DDRi treatment in general (e.g., [48]), synthetic lethality approaches [49] may evolve in clinical practice. Thus, the individual molecular background of bladder cancer patients, including biallelic *ATR* or *PRKDC* deficiencies, should be considered in future studies, which may finally allow treatment of patients with a single drug but with comparable efficacy of tumor cell lethality.

## Data Availability

The data that support the findings of this study are available in the manuscript. Further data may be received from the corresponding author upon reasonable request.

## Supplementary Materials

The following supporting information can be downloaded at: https://www.mdpi.com/article/10.3390/biomedicines10061277/s1, Figure S1: IC50 values of ATR and DNA-PK inhibitors with and without ionizing radiation of normal urothelial cells. Figure S2: Comet assay is illustrated to assess radiation-induced DNA damage upon different radiation doses (2 Gy and 8 Gy). Figure S3: Phospho-western blots of ATR activation upon irradiation. Figure S4: Densitometric quantification of western blot-based protein expression and activation (ATR and DNA-PK). Table S1: Detailed information of TP53 mutations found in SCaBER, VMCUB-1 and J82 cells.
