# Supplementary material for: Effective Radiosensitization of Bladder Cancer Cells by Pharmacological Inhibition of DNA-PK and ATR
Source: Biomedicines. 2022 May 30;10(6):1277. doi: 10.3390/biomedicines10061277 (PMC9220184; doi:10.3390/biomedicines10061277)
Supplement: Supplementary file 1 [file biomedicines-10-01277-s001.zip › biomedicines-1715586-supplementary/Supplementary Figure S1.pdf]

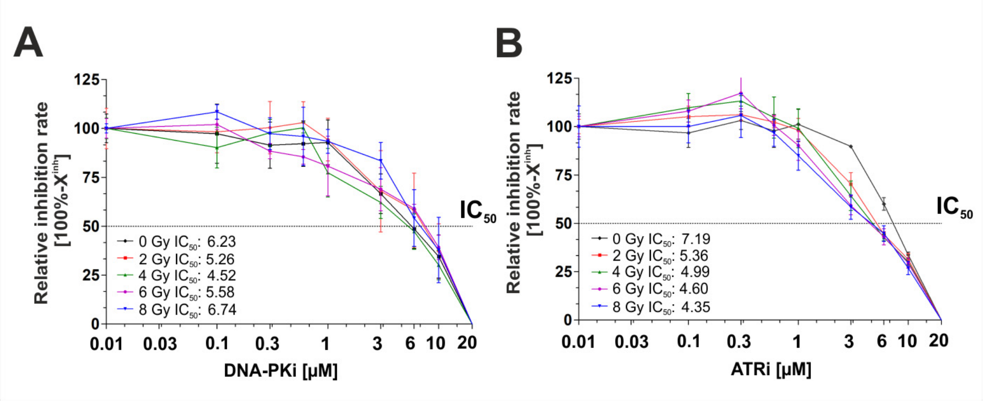

Supplementary Figure S1: (A-B) IC50 values of ATR and DNA-PK inhibitors with and without ionizing radiation of the normal urothelial cell line UROtsa. Short-term single drug response analyses applying DNA-PK and ATR inhibitors upon different radiation doses (0 Gy, 2 Gy, 4 Gy, 6 Gy, 8 Gy) are illustrated. Semi-logarithmic plots show drug response curves (relative inhibition rate = 100% - Xinh). The drug response was determined using XTT assay following 72h incubation with indicated drug concentrations and radiation doses. Dotted line and stated relative IC50= drug concentration causing 50% inhibition. Drug response curves represent means from at least n=3 independent experiments.
