# Supplementary material for: Effective Radiosensitization of Bladder Cancer Cells by Pharmacological Inhibition of DNA-PK and ATR
Source: Biomedicines. 2022 May 30;10(6):1277. doi: 10.3390/biomedicines10061277 (PMC9220184; doi:10.3390/biomedicines10061277)
Supplement: Supplementary file 1 [file biomedicines-10-01277-s001.zip › biomedicines-1715586-supplementary/Supplementary Figure S2.pdf]

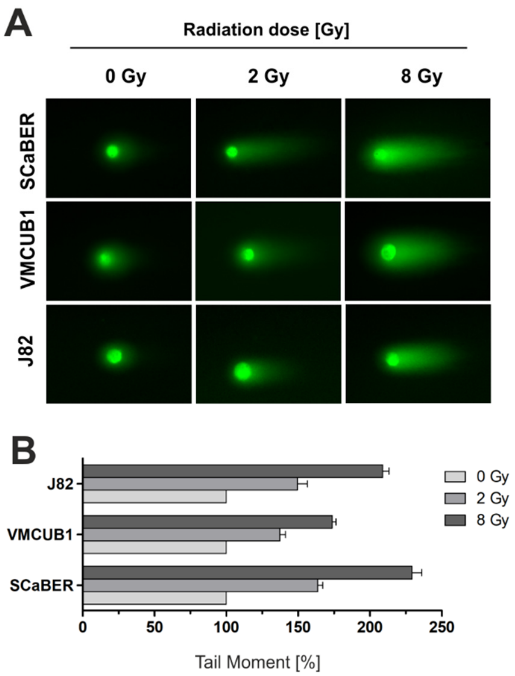

Supplementary Figure S2: A comet assay was performed to assess radiation induced DNA damage upon different radiation doses (2 Gy and 8 Gy). Cells without irradiation were used as control. **(A)** Representative immunofluorescence images of stained nuclear tails are shown for each cell line and radiation dose. **(B)** Semi-quantitative analyses of the Tail Moment upon irradiation for SCaBER, VMCUB-1 and J82 cells. Overall n= 1496 individual cells with corresponding tails were analyzed by image j. The 0 Gy control was used for normalization.
