# Supplementary material for: Effective Radiosensitization of Bladder Cancer Cells by Pharmacological Inhibition of DNA-PK and ATR
Source: Biomedicines. 2022 May 30;10(6):1277. doi: 10.3390/biomedicines10061277 (PMC9220184; doi:10.3390/biomedicines10061277)
Supplement: Supplementary file 1 [file biomedicines-10-01277-s001.zip › biomedicines-1715586-supplementary/Supplementary Figure S3.pdf]

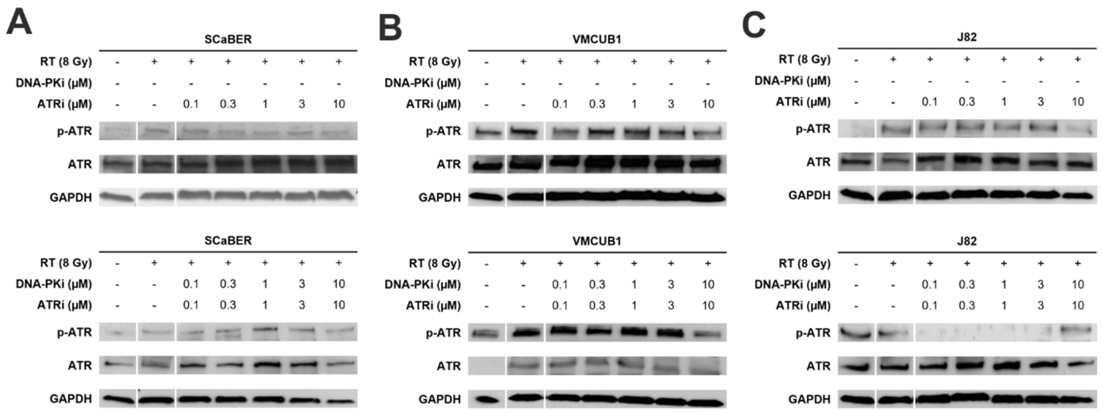

Supplementary Figure S3: Phospho-western blots were performed to assess activation of ATR upon irradiation (radiation dose = 8Gy) as well as single and dual DNA-PK/ATR inhibition in SCaBER (A), VMCUB-1 (B) and J82 (C) bladder cancer cells. ATR phosphorylation at the position Thr1989 was not blocked upon single/dual treatment of increased concentrations of AZD7648 and ceralasertib.
