# Supplementary material for: Effective Radiosensitization of Bladder Cancer Cells by Pharmacological Inhibition of DNA-PK and ATR
Source: Biomedicines. 2022 May 30;10(6):1277. doi: 10.3390/biomedicines10061277 (PMC9220184; doi:10.3390/biomedicines10061277)
Supplement: Supplementary file 1 [file biomedicines-10-01277-s001.zip › biomedicines-1715586-supplementary/Supplementary Figure S4.pdf]

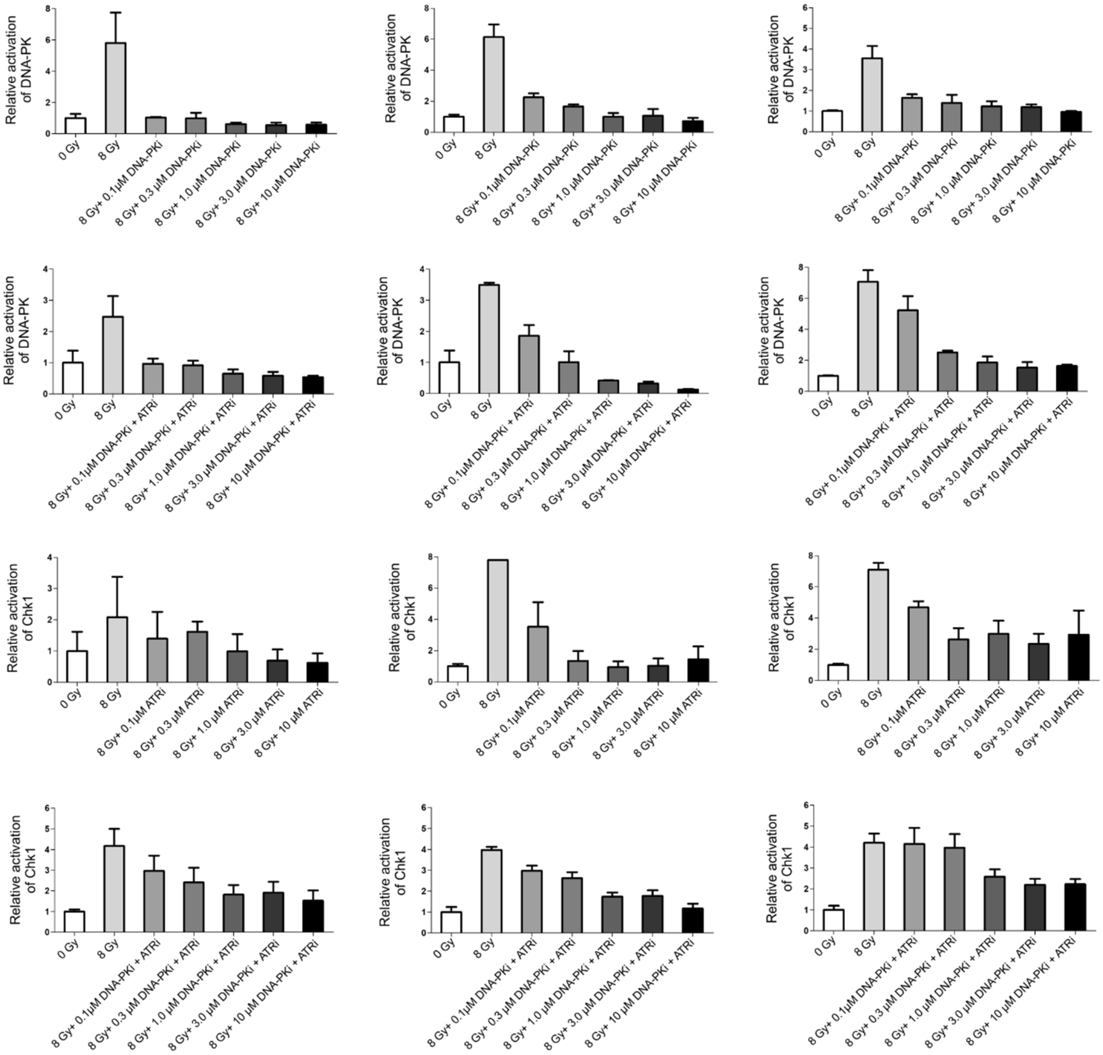


**Supplementary Figure S4:** Densitometric quantification of western blot-based protein expression and activation (ATR and DNA-PK). After standardization by loading control (GAPDH), the relative activation of DNA-PK and CHK1 (p-DNA-PK respectively p-CHK1) was determined by normalizing to the total amount of protein (total DNA-PK respectively total CHK1). Densitometric data represent means from at least n=3 independent experiments.
